# Evidential meta-model for molecular property prediction

**DOI:** 10.1093/bioinformatics/btad604

**Published:** 2023-10-17

**Authors:** Kyung Pyo Ham, Lee Sael

**Affiliations:** Department of Artificial Intelligence, Ajou University, Suwon 16499, Republic of Korea; Department of Artificial Intelligence, Ajou University, Suwon 16499, Republic of Korea; Department of Software and Computer Engineering, Ajou University, Suwon 16499, Republic of Korea

## Abstract

**Motivation:**

The usefulness of supervised molecular property prediction (MPP) is well-recognized in many applications. However, the insufficiency and the imbalance of labeled data make the learning problem difficult. Moreover, the reliability of the predictions is also a huddle in the deployment of MPP models in safety-critical fields.

**Results:**

We propose the Evidential Meta-model for Molecular Property Prediction (EM3P2) method that returns uncertainty estimates along with its predictions. Our EM3P2 trains an evidential graph isomorphism network classifier using multi-task molecular property datasets under the model-agnostic meta-learning (MAML) framework while addressing the problem of data imbalance.

Our results showed better prediction performances compared to existing meta-MPP models. Furthermore, we showed that the uncertainty estimates returned by our EM3P2 can be used to reject uncertain predictions for applications that require higher confidence.

**Availability and implementation:**

Source code available for download at https://github.com/Ajou-DILab/EM3P2.

## 1 Introduction

Molecular property prediction (MPP) is a fundamental task in various industrial fields. Especially in drug discovery, an accurate and trustworthy computational method for MPP can greatly accelerate the process of drug candidate discovery. With the advent of big data and deep neural networks (DNNs), MPP models have improved dramatically. Earlier DNN models have used SMILES (Simplified Molecular Input Line Entry System) encoding (Wieder *et al.* 2020) as input. Today, the input is mostly graph-based, as it is more detailed compared to SMILES. Many methods use a simple graph representation of whole molecules as surveyed in Ham *et al.* (2022). Others use the whole molecular graph with motif graphs (Zhang *et al.* 2021, Zang *et al.* 2023) or mixtures of representations (Jiang *et al.* 2022, Wang *et al.* 2022).

Most current MPP models are based on Graph Neural Networks (GNNs). GNNs take the graph structure, i.e. the natural 2D structure of whole molecules or molecular motifs, as input to neural network models and make supervised predictions. GNNs have been shown to be strong at capturing the features of the nodes and the relationships between individual nodes, making them a powerful tool for MPP. For this reason, over 80 MPP tasks have used variants of GNN (Wieder *et al.* 2020). Among the most widely used GNN models on MPP are the variants of graph neural networks with a message passing scheme, as reviewed in Ham *et al.* (2022). More details on related works are provided in the Supplementary Materials.

However, supervised GNNs, due to their large number of parameters, inherently require a large amount of labeled data. With redundancy removed, labeled data for MPP is often insufficient and highly imbalanced in labels. Moreover, training large models such as GNNs on limited and imbalanced data requires additional measurements for the trustworthiness of the prediction.

To address the data scarcity problem, various few-shot learning-based methods have been proposed for general purposes (Hospedales *et al.* 2021, Crisostomi *et al.* 2022). The goal of few-shot learning is to train a model with only a few labeled data. Few-shot methods are often combined with various other learning methods, such as meta-learning, to improve performance. The goal of meta-learning is to learn a model of models. This model is often easily transferable to specific tasks or domains. In the optimization perspective, when few-shot and meta-learning are combined, the goal becomes learning an easily transferable model that can adapt to new data and tasks with only a few training data (Finn *et al.* 2017). The goal of few-shot learning-based meta-learning fits well with the MPP tasks. That is, we want a model that is trained on various molecule and MPP task pairs to work well on new molecules or new MPP tasks after a simple adaptation with only a few data.

In addition to prediction accuracy, the trustworthiness of prediction results is another critical factor in MPP. This seems even more critical in a label-imbalanced setting, where a model is overly confident in predicting the class label with more samples. Although few-shot meta-learning alleviates the need for large datasets, it does not address the gap between the confidence, i.e. class probability, and the actual accuracy. The trustworthiness of the prediction results can be estimated by the certainty of the model in making the prediction. If an MPP model can return the uncertainty of its prediction, we can better rely on the model and plan for postanalysis. Uncertainty estimation methods approximate model uncertainties to measure the confidence of models in their prediction for a given input. Note that, although uncertainty estimation models provide uncertainty values for each prediction they make, the predicted class probabilities may still be biased toward the majority class.

In this work, we propose a novel meta-MPP classification model called Evidential Meta-model for Molecular Property Prediction (EM3P2) that returns the uncertainty estimate for each model prediction, is able to adapt to new tasks with few labeled data, and is less sensitive to data imbalance. We list our contributions as follows:

- **New method**: We propose a novel uncertainty-aware few-shot meta-learning model, i.e. EM3P2, for molecular property prediction that addresses the data imbalance problem and makes uncertainty adaptions.
- **Comparative analysis**: We compare the performance of EM3P2 with three state-of-the-art few-shot meta-learning models and two representative metric learning models for molecular property prediction and show improved prediction performance.
- **Uncertainty analysis**: We show that uncertainty estimates returned by EM3P2 can be used to determine the trustworthiness of model predictions. We also show that uncertainty estimates can be used as thresholds to improve accuracy.

## 2 Materials and methods

**Figure 1.**
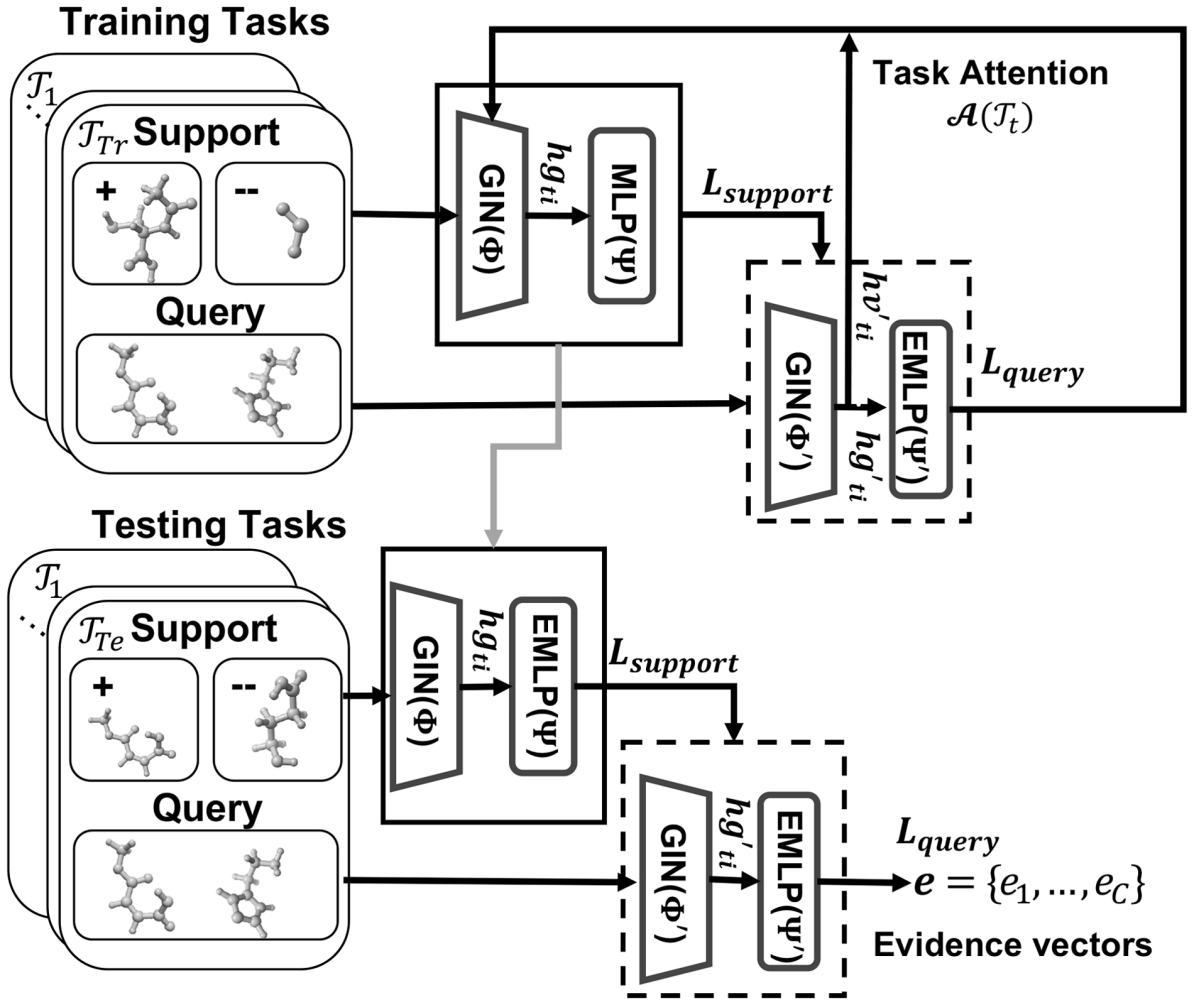
The proposed EM3P2.

**Overview.** Our proposed method, i.e. Evidential Meta-model for Molecular Property Prediction (EM3P2), is illustrated in Fig. 1. Our EM3P2 uses the Model Agnostic Meta-Learning (MAML) (Finn *et al.* 2017, Guo *et al.* 2021) framework with our query balancing technique and evidential adaptations. The EM3P2 consists of a meta-training phase (top) and a meta-testing phase (bottom). In meta-training, the current meta-model is locally adapted with support sets (top bold box), and the adapted model (top dotted box) is used to update the meta-model. In meta-testing, test-task-specific local adaptation is performed with the support sets (bottom bold box), and the adapted model (bottom dotted box) is used to predict the evidence vectors. The components of the EM3P2 are the Graph Isomorphism Networks (GIN) (Xu *et al.* 2019) for graph embedding, the Multi-Layer Perceptron (MLP) for local classifications in the training phase, and the Evidential Multi-Layer Perceptron (EMLP) (Sensoy *et al.* 2018) for evidential meta-classifications. The choice of using MLP instead of EMLP for local adaptation in the training phase is to avoid learning uncertainty values that are specific to each training task.

In the following detailed description of our method, we first describe the overall data-balanced meta-learning framework, then our graph embedding and classification models, and finally the loss functions and regularization terms.

Algorithm 1. EM3P2 Meta-Training

**Require:** Support dataset:$S_{t}=\{x_{i},y_{i}\}$ and query dataset:

  $Q_{t}=\{x\prime_{i},y\prime_{i}\}$ for training task $T_{t}$ for t=1,…,T.

**Require:** Outer-loop, inner-loop learning rate *β*, *α*:

  1: Randomly initialize meta-model parameters Θ={Φ,Ψ}

  2: **while** not done **do**

  3:  **for** each $T_{t}$**do**

  4:   sample *N* support data from $S_{t}$

  5:   **for**i=1,…,N**do**

  6:    $hg_{ti},\_\_=\text{GIN}(x_{ti},\Phi )$

  7:    ${\hat{y}}_{ti}$ = MLP$\left(hg_{ti},\Psi \right)$

  8:   **end for**

  9:   $L_{t}\leftarrow$ Cross-Entropy Loss with $\left\{{\hat{y}}_{t1},\ldots ,{\hat{y}}_{tN}\right\}$

10:   $\Theta \prime_{t}=\Theta -\alpha \nabla_{\Theta }L_{T_{t}}$ ▹ Eq.(1)

11:   sample *M*/*C* number of query data from each *C* classes       from $Q_{t}$ for meta update

12:   **for**i=1,…,M**do**

13:    $hg\prime_{ti},hv\prime_{ti}=\mathrm{GIN}(x\prime_{ti},\Phi_{t}^{\prime })$

14:    $\hat{y}\prime_{ti},e\prime_{ti}$ = EMLP$\left(hg\prime_{ti},\Psi_{t}^{\prime }\right)$

15:   **end for**

16:   $L\prime_{t}\leftarrow$Eq.(14) with $\left\{\left(\hat{y}\prime_{t1},e\prime_{t1}),\ldots ,(\hat{y}\prime_{tM},e\prime_{tM}\right)\right\}$

17:   $hv\prime_{t}=\text{MEAN}\left(hv\prime_{t1},\ldots ,hv\prime_{tN}\right)$

18:  **end for**

19:  $\left\{A\left(T_{1}\right),\ldots ,A\left(T_{T}\right)\right\}\leftarrow$Eq.(2) with $\left\{hv\prime_{1},\ldots ,hv\prime_{T}\right\}$

20:  $\Theta =\Theta -\beta \nabla_{\Theta }\sum_{t=1}^{T}A\left(T_{t}\right)\cdot L\prime_{T_{t}}$ ▹ Eq.(3)

21: **end while**

### 2.1 Evidential meta learning framework

Our EM3P2 has two phases, i.e. meta-training and meta-testing, similar to MAML (Finn *et al.* 2017), with an added query balancing technique and design choices for evidential adaptations.

### 2.2 EM3P2 *meta training*

The goal of meta-training is to obtain a meta-model that is easily adaptable to new data and tasks. Algorithm 1 describes the overall meta-training process of our EM3P2. The meta-training phase iteratively selects a training task $T_{t}$ from $T_{\mathrm{train}}$ (lines 3), makes a local task adaptation with the selected support dataset (lines 4–10), computes the evidential query loss and the average node embedding using the locally adapted model (Equation 14) with the selected query dataset (lines 11–18), and uses the query losses and average node embedding to make a task-attended update for the parameters of the evidential meta-model (lines 19–20).

**Evidential task-adaption.** More specifically, in the local adaptation of the meta-model, for *t*th task $T_{t}$, the current evidential meta-model with parameter Θ={Φ,Ψ} is adapted to the task-specific local model using *N* number (*N*-shot where N=1,10 for our test) of support data points $x_{ti}\in S_{t}$. First, the graph embedding vector $hg_{ti}$ is obtained by running the GIN model (Sec.) with the current meta-GIN parameter Φ on the support data $x_{ti}$ (line 6). Then, the graph embedding vector $hg_{ti}$ is used to run the multi-layer perceptron (MLP) with the parameter set Ψ to obtain a prediction ${\hat{y}}_{ti}$ (line 7). After obtaining the results for the selected support data points, a support loss ($L_{t}$), which is simply a cross-entropy loss, is computed (lines 9). Note that choosing not to learn the evidential parameters, i.e. not using EMLP, for the local adaptations in the training phase is an important choice to avoid biasing the prediction confidence of the meta-model on specific training tasks. The support loss is used to update the local model parameters $\Theta_{t}^{\prime }=\{\Phi_{t}^{\prime },\Psi_{t}^{\prime }\}$ (lines 10) as follows:

(1) $$\Theta_{t}^{\prime }=\Theta -\alpha \nabla_{\Theta }L_{T_{t}}(MLP_{\Psi }(GIN_{\Phi })).$$

**Query balancing (QB).** The next step is to sample *M* number of data points from the query dataset $Q_{t}=\{x\prime_{i},y\prime_{i}\}$ for use in the evidential meta-model update. Under the default MAML setting, the queries are randomly selected from the query dataset. However, in a highly imbalanced environment, random sampling results in the training of a meta-model that always predicts in the direction of the majority class. To properly train our EM3P2 under data imbalance, when selecting *M* data points, we additionally ensure that the balanced number of samples are selected from each of the *C* numbers of classes by selecting *M*/*C* of query data points from each class (lines 11).

**Evidential meta-model update.** For each of the sampled query data $x\prime_{ti}$, graph embedding vectors $hg\prime_{ti}$ and node embedding vectors $hv\prime_{ti}$ are computed using the task-adapted GIN parameters $\Phi \prime_{t}$. Graph embedding vectors $hg\prime_{ti}$ are used to compute class predictions $\hat{y}\prime_{ti}$ and evidence vectors $e\prime_{ti}$ using the task-adapted classifier, i.e. evidential multi-layer perception (EMLP) (lines 12–15). The class predictions and the evidence vectors of the sampled query dataset are used to obtain the evidential query loss (line 16).

The final step of the iteration is to perform a task attended parameter update for the evidential meta-model. The average node embedding for each of the training tasks $hv\prime_{t}$ (line 11) is used to compute the task attention values for each task $A(T_{t})$ for t=1,….T (line 19). The task attention values are calculated as follows (Guo *et al.* 2021):

(2) $$A(T_{t})=\frac{\;\text{exp}(\text{MLP}(hv_{T_{t}}))}{\sum_{T_{t}^{\prime }\in T}\;\text{exp}(\text{MLP}(hv_{T_{t}^{\prime }}))},$$

where $hv_{T_{t}}=$ MEAN$\left({\left\{hv_{T_{t}i}\right\}}_{i=1}^{N}\right)$ and MLP is a single layer of a multi-layer perceptron. The evidential query losses $L\prime_{T_{t}}$ and the attention values $A(T_{t})$ are then used to update the evidential meta-model (line 20) as follows:

(3) $$\Theta =\Theta -\beta \nabla_{\Theta }\sum_{t=1}^{T}A\left(T_{t}\right)\cdot L\prime_{T_{t}}(\mathrm{EML}P_{\Psi_{t}^{\prime }}(GIN_{\Phi_{t}^{\prime }})).$$

Details of the model components, GIN, MLP, EMLP, and loss functions are described in the later sections.

Algorithm 2. EM3P2 Meta-Testing

**Require:** *N* support data $s_{t}=\{x_{i},y_{i}\}$ and query data $q_{t}=\{x\prime \}$     of *t*th testing task $T_{t}$

**Require:** meta-model parameters Θ={Φ,Ψ}

1: $\Theta \prime_{t}\leftarrow \Theta$

2: **while** not done **do**

3:  **for**i=1,…,N**do**

4:   ${\hat{y}}_{ti}$ = EMLP$\left(\text{GIN}(x_{ti},\Phi \prime_{t}),\Psi \prime_{t}\right)$

5:  **end for**

6:  $L_{t}\leftarrow$Eq.(14) with $\left\{{\hat{y}}_{t1},\ldots ,{\hat{y}}_{tN}\right\}$

7:  $\Theta \prime_{t}=\Theta \prime_{t}-\alpha \nabla_{\Theta \prime }L_{T_{t}}$ ▹ Eq.(4)

8: **end while**

9: $\hat{y}\prime ,e\prime$ = EMLP(GIN(x′,Φ′),Ψ′)

### 2.3 EM3P2 *meta testing*

The meta-testing phase is similar to the meta-training phase without the meta-model update. The entire testing process is described in Algorithm 2. We assume a new query data $q_{t}=\{x\prime \}$ under a new task $T_{t}$. We also assume that there is *N* number of support data with labels under the same task. The learned evidence model with parameters Θ={Φ,Ψ} is adapted to the new task $T_{t}$ under the evidence model setting (lines 1–4). The support loss (line 5) is used to update the local model parameters Θ′={Φ′,Ψ′} (lines 6) as follows:

(4) $$\Theta \prime =\Theta \prime -\alpha \nabla_{\Theta \prime }L_{T_{t}}(\mathrm{EML}P_{\Psi \prime }(GIN_{\Phi \prime })).$$

The final adapted model is used to obtain the prediction label and the evidence vector (lines 7–8) for evaluation.

### 2.4 Graph embedding and classification model

Our method uses the Graph Isomorphism Network (Xu *et al.* 2019) for molecular graph and node embedding and the Evidential Multi-layer Perceptron (Sensoy *et al.* 2018) for molecular property classification.

### 2.5 Graph isomorphism network (GIN)

Graph Isomorphism Network (GIN) (Xu *et al.* 2019) is a recently introduced graph neural network that has been proven to have equal power to the Weisfeiler-Lehman test under mild conditions. GIN is a type of message-passing neural network that utilizes multi-layer perceptrons (MLP) to aggregate and combine node representations from each node and its neighbor within the *K*-hops. More specifically, a *K* layered GIN, given a graph G=(V,E) and initial node vectors **X**_*v*_, compute the representation of node *v* at *k*th layer as follows:

(5) $$h_{v}{}^{\left(k\right)}={\text{MLP}}^{\left(k\right)}\left((1+\epsilon^{\left(k\right)})\cdot h_{v}{}^{\left(k-1\right)}+\sum_{u\in N_{\left(v\right)}}h_{u}^{\left(k-1\right)}\right)$$

where *N*(*v*) is a set of neighbors of node *v*, and $h_{v}{}^{\left(0\right)}=X_{v}$. After the final layer, the final graph and node representations for classification are obtained from the readouts of the last layer *l*. More specifically, a node embedding is computed as $h_{v}=\{h_{v}^{\left(l\right)}|v\in V\}$ and a graph embedding as $h_{g}=\text{MEAN}(h_{v}^{\left(l\right)}|v\in V),$ where *MEAN(.)* function averages all node embedding into the graph. The resulting graph embedding $h_{g}$ is used as input to the classification layers.

### 2.6 Evidential multi-layer perceptron (EMLP)

Among various uncertainty estimation methods, evidential neural networks (ENN) show comparable results to others with less number of parameters and less inference time complexity (Sensoy *et al.* 2018, Ham *et al.* 2022). ENN is based on the observation that softmax values, typical results of classification models, have the problem of inflating predicted class probabilities due mostly to the use of exponential terms. Instead of the typical softmax parameters set of a categorical distribution, the ENN for classification models (Sensoy *et al.* 2018) replaces the parameters with that of a Dirichlet distribution. The Dirichlet distribution for *C* class probabilities given parameter vector $\alpha \in R^{C}$ can be written as follows:

(6) $$D(p|\alpha )=\{\begin{array}{cc} \frac{1}{B(\alpha )}\prod_{i=1}^{C}p_{i}^{\alpha_{i}-1} & \;\text{for}\;p\in S_{C},\\ 0 & \;\text{otherwise},\; \end{array}$$

where $S_{C}=\{p|\sum_{j=1}^{C}p_{j}$ and $0\leq p_{1},\ldots ,p_{C}\leq 1\}$B(α) are the *C*-dimensional unit simplex and the multinomial beta function, respectively. This allows the model to represent the predictions of the model as a distribution over possible classes instead of softmax point estimates.

For our EM3P2 model, graph embedding outputted by GIN goes through an evidential multi-layer perception (EMLP) where the last layer is an activation layer (we used ReLU), instead of a typical softmax layer. The result of the activation layer is taken as the evidence vector $e\in R_{+}^{C}$.

### 2.7 Estimating uncertainty

The class probabilities, confidence, and uncertainty of the prediction can be inferred from the evidence vector **e**_*i*_, for the input **x**_*i*_, according to the Dempster-Shafer theory of evidence (Dempster 2008) and subjective logic (Jøsang 2016). That is, the relation between the total evidence $e_{c}\geq 0$ for class *c* and the Dirichlet parameter $\alpha_{c}\geq 1$ is $\alpha_{c}=e_{c}+1$. Also, the prediction uncertainty u≥0 has the following relationship to the evidence: $u+\sum_{c=1}^{C}e_{c}/(\sum_{j=1}^{C}e_{j}+1)=1.$ With the two equations and the prediction result of the positive evidence vector **e**_*i*_ of size *C* for the input **x**_*i*_, we can extract the class probability as

(7) $${\hat{p}}_{ic}=\frac{e_{ic}+1}{\sum_{j=1}^{C}e_{ij}+1},$$

the confidence as

(8) $${\hat{p}}_{i}=\text{max}({\hat{p}}_{ic}),$$

and the prediction uncertainty as

(9) $$u_{i}=\frac{C}{\sum_{j=1}^{C}e_{ij}+1}.$$

If the estimated uncertainty *u_i_* is high, the prediction $\hat{y}$ may not be trustworthy (Sensoy *et al.* 2018, Ham *et al.* 2022).

### 2.8 Loss functions

There are three components to the overall loss function of our proposed EM3P2. First, we describe the evidential loss functions used. Then, we describe two regularizers used to improve uncertainty estimation and model calibration.

### 2.9 Evidential loss function

Our EM3P2 uses the Dirichlet cross-entropy loss (Sensoy *et al.* 2018) as the base loss function of EMLP. Note that for MLP, we use the regular cross-entropy loss. The Dirichlet cross-entropy loss is as follows (Sensoy *et al.* 2018):

(10) $$\begin{array}{c} L_{\mathrm{dce}}(x_{i},y_{i},\Theta )=\int \left[\sum_{j=1}^{C}-y_{ij}\;\text{log}(p_{ij})\right]\frac{1}{B(\alpha_{i})}\prod_{j=1}^{C}p_{ij}^{\alpha_{ij}-1}dp_{i}\\ =\sum_{j=1}^{C}y_{ij}\left(\psi (\sum_{c=1}^{C}\alpha_{ic})-\psi (\alpha_{ij}))\right) \end{array}$$

where ψ(·) is a *digamma* function.

### 2.10 Belief regularizer (BR)

Belief regularization penalizes false beliefs, which are known to exist in few-shot models (Pandey and Yu 2022). If the false belief can be accurately quantified, it can be used to guide the model in training to minimize false predictions. The belief error is expressed as the sum of the evidence values for false classes for an input. The belief regularization can then be expressed as (Pandey and Yu 2022):

(11) $$L_{br}(\Theta )=\frac{1}{N}\sum_{i}^{N}||e_{i}⊙(1-y_{i})|{|}_{1},$$

where $e_{i}⊙(1-y_{i})$ is an element-wise product of the evidence vector $e_{i}$ and the one-hot vector representation of the class label $y_{i}$ for input $x_{i}$.

### 2.11 Accuracy versus uncertainty loss regularizer (AvUC)

The accuracy versus uncertainty (AvUC) measures the calibration of the model on its certainty of predictions and the actual accuracy as follows (Guo *et al.* 2017, Krishnan and Tickoo 2020, Bao *et al.* 2021):

(12) $$\mathrm{AvUC}=\frac{n_{AC}+n_{IU}}{n_{AC}+n_{AU}+n_{IC}+n_{IU}},$$

where the *n_AC_*, *n_AU_*, *n_IC_*, and *n_IU_* are the numbers of results in the four categories: accurate and certain (AC), accurate and uncertain, inaccurate and certain (IC), and inaccurate and uncertain (IU). The AvUC regularizer puts a constrain on the cross entropy between maximum class probability *p_i_* and uncertainty *u_i_* of data **x**_*i*_ as follows (Bao *et al.* 2021):

(13) $$\begin{array}{c} L_{\mathrm{AvUC}}(\Theta ,\lambda_{c})=-\lambda_{c}\sum_{i\in \left\{\hat{y_{i}}=y_{i}\right\}}p_{i}\;\text{log}(1-u_{i})\\ -(1-\lambda_{c})\sum_{i\in \left\{\hat{y_{i}}\neq y_{i}\right\}}\left(1-p_{i}\right)\;\text{log}(u_{i}), \end{array}$$

where *λ_c_* is the strength of the constraint.

### 2.12 Overall loss function

Two losses are used for local adaptation and meta-model updates. For the local adaptation of the training phase (MLP updates), the regular cross-entropy loss is used, i.e. $\sum_{i}^{N}\sum_{j}^{C}y_{ij}\;\text{log}\;p_{i}j$. For the rest of the model updates (EMLP updates), the following loss is used:

(14) $$\begin{array}{c} L_{\mathrm{EMLP}}(\Theta )=L_{\mathrm{dce}}(\Theta )+\lambda_{b}L_{br}(\Theta )+L_{\mathrm{AvUC}}(\lambda_{c}) \end{array}$$

where $\lambda_{b},\lambda_{c}$ are annealing factors that increase monotonically with the training epoch increases. In our training, each annealing factor is defined as $\lambda_{b}=\lambda_{0}t/T$ and $\lambda_{c}=0.1*\lambda_{0}t/T$. Each annealing factor increases from *λ*_0_ to 1.0 and 0.1, respectively, we set a smaller scale for *λ_b_* with the observation that the model becomes more sensitive to calibration when the training dataset is small. In the early stages of training, the model focuses more on learning the data and acquiring evidence, but as training progresses, the model gradually penalizes false beliefs and false predictions with respect to accuracy.

## 3 Experiments

**Dataset.** We evaluated our experiment on the Tox21, SIDER, MUV datasets provided by MoleculeNet (Wu *et al.* 2018). A summary of the datasets is provided in the Table 1. Tox21 is a public database that measures the toxicity of molecules used in the 2014 Tox21 Data Challenge http://tripod.nih.gov/tox21/challenge/. The dataset contains information on whether or not each of the 7831 molecules binds to the 12 different targets. Tox21 is a highly imbalanced dataset, with more than twelve times more negative labels than positive labels (Table 1 last row). We considered predicting binding to each of the 12 targets as an independent task, resulting in 12 different tasks for the 7831 molecules. The Side Effect Resources (SIDER) is a database of 1427 marketed drugs and their side effects, categorized into 27 types (Kuhn *et al.* 2016, Altae-Tran *et al.* 2017). The SIDER dataset is somewhat imbalanced with a mix of positively biased, negatively biased, and relatively balanced tasks. The overall ratio of positive to negative labels is 0.76 overall. The 27 side effects were again considered independent tasks, resulting in 27 different tasks for the 1427 molecules. Maximum Unbiased Validation (MUV) dataset, which contains information about whether a molecule binds to a target protein. Each target protein can be considered as an independent task. The MUV contains 93087 molecules and 17 protein targets or tasks. Each task is highly imbalanced with a positive to negative label ratio of 0.002.

**Table 1.** Summary of datasets.

Dataset	Tox21	SIDER	MUV
Molecules	7831	1427	93087
Tasks	12	27	17
Meta-training tasks	9	21	12
Meta-testing tasks	3	6	5
Positive/negative label ratio	0.08	0.76	0.002

The three datasets have molecules written in SMILES strings. We converted the SMILES strings into molecular plots using the Rdkit.Chem (Landrum *et al.* 2013) package to generate the input. For all our experiments, the Tox21 dataset was divided into nine training and three test tasks, the SIDER dataset was divided into 21 training and six test tasks, and the MUV dataset was divided into 12 training and five test tasks. For each task, we randomly preselected support and query molecules as support and query sets, respectively.

**Compared Methods.** We compare our EM3P2 with molecular property prediction (MPP) methods based on meta-learning or metric learning framework.

- MAML (Finn *et al.* 2017) is a task-agnostic algorithm for few-shot meta-learning, where model parameters are trained using a small number of samples. We used the GIN and MLP as the base learner for MAML.
- Pre-GNN (Hu *et al.* 2020) is the MAML model with associated GIN, which uses self-supervised learning to capture local and global information of graph data.
- The Pre-PAR (Wang *et al.* 2021) is similar to the MAML model, but with the added step of generating a relationship graph between molecules with the property-aware embedding of support and query molecules.
- SiameseNet (Koch *et al.* 2015) is a model based on the Siamese network, which is a metric-based few-shot learning algorithm. We used the GIN for molecular representation and measured the similarity between two inputs using the cosine distance. The relationship is considered positive if both input molecules are positive, otherwise negative.
- Protonet (Snell *et al.* 2017, Crisostomi *et al.* 2022) is a model based on a prototypical network, which is also a metric-based few-shot learning algorithm. We used the GIN for molecular representation and cosine distance is used to measure the distance between the prototypes and the query sample.

**Reproducibility Setting.** The experiments were performed on an NVIDIA GeForce GTX 1080 Ti GPU with the following implementation details. MAML was implemented using the learn2learn library. We used author codes for Pre-GNN and Pre-PAR. Both metric-based methods, i.e. SiameseNet and Protonet, were re-implemented. We trained the models using the default settings. For our EM3P2, we used the pretrained GIN of Pre-GNN and implemented the rest by referencing to ENN and Pre-GNN using the Pytorch and Pytorch-Geometric libraries.

### 3.1 Comparison with the state-of-the-art

We compared the prediction performance of our EM3P2 (no uncertainty threshold) with the three existing meta-based MPP methods and two metric-based MPP methods using the area under the receiver operating characteristic curve (ROC-AUC) (Table 2). Overall, our EM3P2 performed the best compared to other methods for both 1-shot and 10-shot cases, except for the 1-shot case on the MUV dataset. We also note that the results for the SIDER dataset are consistently lower for all methods. We suspect that this is due to the categorization of side effects into 27 types, with different granularities and guidelines for each group as well as the mixed positive and negative bias tasks. Results for the MUV dataset also had low prediction performance. This is due to the extremely unbalanced nature of the dataset (with a 0.002 positive-to-negative label ratio). We will show later that using the uncertainty threshold improves the performance of our EM3P2.

**Table 2.** ROC-AUC values of our EM3P2 and compared methods.

DatasetMethod	Tox21		SIDER		MUV	
	1-shot	10-shot	1-shot	10-shot	1-shot	10-shot
MAML	0.621	0.662	0.648	0.648	0.553	0.598
Pre-GNN	0.770	0.767	0.694	0.694	0.628	0.602
Pre-PAR	0.778	0.799	0.691	0.726	0.682	0.642
SiameseNet	0.768	0.773	0.643	0.643	**0.713**	0.651
Protonet	0.542	0.787	0.567	0.718	0.631	0.662

EM3P2^a^	**0.833**	**0.834**	**0.792**	**0.794**	0.637	**0.695**

a Our default proposed model without a uncertainty threshold.

The best performances are in bold face numbers.

### 3.2 Ablation study

Table 3 shows the ROC-AUC and precision values for each variant of our EM3P2. The variants are based on combinations of factors: whether query balancing (QB) or random sampling is used, whether belief regularizer (BR) is used, and whether accuracy versus uncertainty curve regularizer (AvUC) is used. We observed that each of these factors contributes to the overall accuracy of our EM3P2. Overall, the use of QB improves accuracy by at least 23%. The combination of QB and BR improves the precision even more and achieves the best precision values. In terms of prediction ROC-AUC values, the use of all factors resulted in the best performance for the Tox21 and SIDER datasets. For the MUV dataset, due to its extreme bias, the ROC-AUC values were all similar with a difference within 0.008 when using any combination of the three constraints. This can be mitigated by training weights for BR and AvUC. However, for our current work, we have fixed the *lambda* values as described in section. Our EM3P2 combines all three factors in the default setting.

**Table 3.** Ablation studies of EM3P2 reporting ROC-AUC (floating point) and precision (percentage).^a^^,^^b^

QB	BR	AvUC	Tox21		SIDER		MUV	
			1-shot	10-shot	1-shot	10-shot	1-shot	10-shot
✗	✗	✗	0.815	0.821	0.733	0.720	**0.630**	0.552
			11%	8%	41%	43%	0%	0%
✓	✗	✗	0.816	0.817	0.698	0.698	0.628	**0.638**
			62%	54%	73%	68%	23%	30%
✓	✓	✗	0.832	0.833	0.776	0.783	0.629	0.631
			**64%**	**60%**	**76%**	**70%**	**29%**	**40%**
✓	✓	✓	**0.833**	**0.834**	**0.792**	**0.794**	0.629	0.630
			53%	50%	74%	69%	28%	35%

a QB, query balancing in meta training; BR, Belief Regularizer; AvUC, Accuracy Versus Uncertainty Loss Regularizer.

b The precision values are calculated for the minority labels.

The best performances are in bold face numbers.

### 3.3 Uncertainty threshold

**Figure 2.**
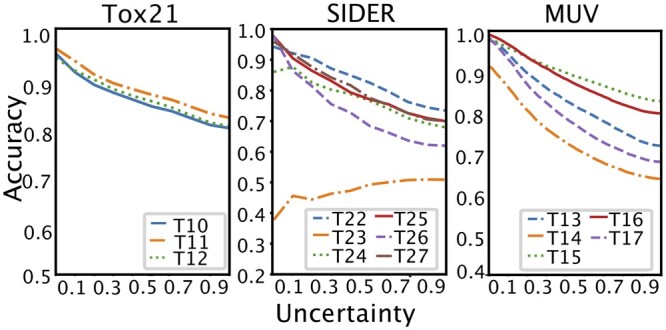
Accuracy according to uncertainty threshold for 10-shot cases.

Figure 2 shows changes in accuracy values with respect to uncertainty. We can see that, with the exception of Task 23 (T23) in SIDER, the test tasks in the Tox21, SIDER, and MUV datasets increase in accuracy as uncertainty decreases. For SIDER task 23, our learned meta-model was not able to adapt properly. We suspect two reasons. First, task 23 is the side effect category for *Pregnancy, puerperium & perinatal conditions*, which is a significantly different categorization from the majority of other tasks that map side effects to system organ classes. This results in overall low accuracy compared to other test tasks. Second, task 23 is positively biased (1302:125) unlike typical tasks that are usually negatively biased. In fact, accuracy increased after a steep decline with increasing uncertainty when we trained our EM3P2 with balanced or positively biased tasks and tested the model with the negatively biased tasks. Additional results for the SIDER dataset can be found in the Supplementary Materials.

With the calibrated accuracy and uncertainty values, our trained EM3P2 was able to predict *I don’t know* for predictions with uncertainty values higher than the threshold. We used the uncertainty values as thresholds for whether or not to accept the prediction. Table 4 shows the average accuracy according to the uncertainty thresholds (*ut*) in 1- and 10-shot settings.

**Table 4.** Accuracy of EM3P2 with uncertainty threshold.

Dataset	Tox21		SIDER		MUV	
Method	1-shot	10-shot	1-shot	10-shot	1-shot	10-shot
EM3P2 ut≤0.1	**93.5**	**92.1**	**84.5**	**86.8**	**97.4**	**97.7**
EM3P2 ut≤0.2	90.6	89.6	80.2	82.1	92.9	92.4
EM3P2 ut≤0.3	88.7	87.4	75.3	79.0	89.8	88.7
EM3P2 ut≤0.4	87.3	86.2	72.9	75.6	87.6	85.9
EM3P2 no threshold	82.1	81.6	64.3	66.1	79.8	76.6

The best performances are in bold face numbers.

### 3.4 Comparison of EMLP and MLP empirical results

To show that quantified evidence provides better confidence, we looked at individual test cases in detail. Here, we compared individual results when only multi-layer perceptrons (MLPs) were used for classification and when evidential multi-layer perceptrons (EMLPs) as in our default setting. Table 5 shows the prediction results for captafol molecule in the Tox21 dataset. The true labels for the test tasks were all positive. However, the model using MLP classification predicted negative with a high class probability, while our EM3P2 using EMLP returned high uncertainty values and predicted *I don’t know (?)*. More empirical results for SIDER and MUV datasets are provided in the Supplementary Materials.

**Table 5.** Detailed test task result of captafol in Tox21 dataset.

	Measure	Task10	Task11	Task12
EM3P2	+ Evidence	0.382	0.283	0.255
	Uncertainty	0.672	0.717	0.745
	Prediction	?	?	?
EM3P2 (MLP)	– Class prob.	0.819	0.816	0.815
	Prediction			

## 4 Conclusion

We have proposed a Evidential Meta-model for Molecular Property Prediction (EM3P2) method for evidence-aware molecular property prediction (MPP). This method is capable of learning from few and often imbalanced data. The EM3P2 is a novel uncertainty-aware few-shot meta-learning model for MPP that provides uncertainty estimates for each model prediction. It adapts well to novel tasks with limited labeled data and is less sensitive to data imbalance. In addition to having comparable prediction performance to other few shot MPP models, we can use the uncertainty threshold to obtain better and more confident predictions. Overall, the proposed EM3P2 model and our analysis of the uncertainty estimation to the MPP dataset provide valuable insights and advances in addressing the challenges of data imbalance and reliability in MPP. Our EM3P2 shows the potential of MPP models to accelerate the identification of potential drug candidates and to improve reliability in sensitive areas.

## Supplementary Material

btad604_Supplementary_Data

## Data Availability

The data underlying this article were accessed from MoleculeNet, https://moleculenet.org/.
