# Supplementary material for: Evidential meta-model for molecular property prediction
Source: Bioinformatics. 2023 Oct 17;39(10):btad604. doi: 10.1093/bioinformatics/btad604 (PMC10597608; doi:10.1093/bioinformatics/btad604)
Supplement: btad604_Supplementary_Data [file btad604_supplementary_data.pdf]

Bioinformatics, 2023, pp. 1–7

doi: DOI HERE
Advance Access Publication Date: Day Month Year
Supplementary Materials

SUPPLEMENTARY MATERIALS

Supplementary Materials for Evidential Meta-model for Molecular Property Prediction

Kyung Pyo Ham[1] and Lee Sael[2,*]

[1]Department of Artificial Intelligence, Ajou University, 16499, Suwon, South Korea and [2]Dept. of Software and Computer Engineering, Ajou University, 16499, Suwon, Republic of Korea
[*]Corresponding author. sael@ajou.ac.kr
FOR PUBLISHER ONLY Received on Date Month Year; revised on Date Month Year; accepted on Date Month Year

Here we provide supplementary materials and supplementary results.

S1: Related Works

Computational MPP, which can be divided into the pre-deep neural network era and the post-deep neural network era, has long been of great interest. Before the advent of big data and deep neural networks (DNNs), the main concern was the design of fingerprints and input features of molecules, which were regionally biased and less extensible to novel molecules [8]. The advent of DNN has allowed researchers to put less effort into the extraction of features and more effort into the design of the learning models. The advent of big data and deep neural networks (DNNs) allowed researchers to put less effort into the extraction of features and more effort into the design of the learning models.

The inputs to MPP models for DNN models can be summarized as follows. Earlier DNN models have used SMILES (Simplified Molecular Input Line Entry System) encoding [8] as input. Most current MPP models take molecular graphs as input and utilize Graph Neural Networks (GNNs).

Most well known few-shot MPP models are combined with meta-models or with metric models. Meta-learning aims to learn a model of models using multiple datasets. Metric learning focuses on learning an embedding space, moving similar elements closer together and dissimilar elements further apart in the transformed space. In the following, we list and describe three few-shot meta-models and two few-shot metric models.

- **MAML** [1] optimizes the initial parameters of a model in order to quickly adapt it to new tasks. MAML enables the model to perform well in multi-task, few-shot scenarios by fine-tuning these parameters with a few gradient steps on new tasks.
- **Pre-GNN** [2] has emerged as a notable technique for optimization-based few-shot and meta-learning involving graph-structured data. It involves pre-training a graph neural network on various tasks under the MAML framework, thereby enhancing its ability to generalize to new tasks involving graph data.
- **Property-aware Relation Network (PAR)** [7] uses negative and positive examples to obtain the property-aware embedding function to transform the generic molecular embeddings into substructure-aware space. Then, to estimate the relation graph between the few-shot examples, an adaptive relation graph learning module is generated. The whole process is trained under a meta-learning approach.
- **Siamese Networks**[3] have been extensively studied for similarity learning tasks in a one-shot learning setting. In the training process, two inputs are processed through shared weights, allowing the network to learn a distance metric that measures the similarity between pairs of instances. If both inputs are positive, the relationship is considered positive or similar, otherwise negative.
- **Prototypical Networks**[6] learns a metric space where instances of the same class are closer to each other, allowing efficient classification with limited labeled data. The model uses prototypes, which are the average of embeddings in the same class, to measure the similarity between the prototypes and a new sample to determine which class the sample belongs to.

S2: Data Summary

© The Author 2023. Published by Oxford University Press. All rights reserved. For permissions, please e-mail: journals.permissions@oup.com

Task	Toxic-effect	Negative	Positive	Unlabeled
Task1	NR-AR	6956	309	566
Task2	NR-AR-LBD	6521	237	1073
Task3	NR-AhR	5781	768	1282
Task4	NR-Aromatase	5521	300	2010
Task5	NR-ER	5400	793	1638
Task6	NR-ER-LBD	6605	350	876
Task7	NR-PPAR-gamma	6264	186	1381
Task8	SR-ARE	4890	942	1999
Task9	SR-ATAD5	6808	264	759
Task10	SR-HSE	6095	372	1364
Task11	SR-MMP	4892	918	2021
Task12	SR-p53	6351	423	1057

Table 1. Data and Task Description of Tox21 dataset.

Task	Side-Effect	Negative	Positive
Task1	Hepatobiliary disorders	684	743
Task2	Metabolism & Nutrition disorders	431	996
Task3	product issues	1405	22
Task4	eye disorders	551	876
task5	Investigations	276	1151
Task6	Musculoskeletal & Connective tissue disorders	430	997
Task7	Gastrointestinal disorders	129	1298
Task8	Social Circumstances	1176	251
Task9	Immune System disorders	403	1024
Task10	Reproductive system & breast disorders	700	727
Task11	Neoplasms benign, malignant & unspecified(incl cysts & polyps	1051	376
Task12	General disorders & administration site conditions	135	1292
Task13	Endocrine disorders	1104	323
Task14	Surgical & medical procedures	1214	213
Task15	Vascular disorders	319	1108
Task16	Blood & lymphatic system disorders	542	885
Task17	Skin & subcutaneous tissue disorders	109	1318
Task18	congenital, familial & genetic disorders	1174	253
Task19	Infections & infestations	421	1006
Task20	Respiratory, thoracic & mediastinal disorders	367	1060
Task21	Psychiatric disorders	411	1016
Task22	Renal & urinary disorders	516	911
Task23	Pregnancy, puerperium & perinatal conditions	1302	125
Task24	Ear & labyrinth disorders	768	659
Task25	Cardiac disorders	439	988
Task26	Nervous system disorders	123	1304
Task27	Injury, poisoning & procedural complications	481	946

Table 2. Task Description of SIDER Dataset.

Task	Target	Target	mode of interaction	Target class	Negative	Positive
Task1	MUV-466	s1p1 rec.	agonists	GPCR	14814	27
Task2	MUV-548	PKA	inhibitors	kinase	14705	29
Task3	MUV-600	SF1	inhibitors	nuclear receptor	14698	30
Task4	MUV-644	Rho-Kinase2	inhibitors	kinase	14593	30
Task5	MUV-652	HIV RT-TNase	inhibitors	RNase	14873	29
Task6	MUV-689	Eph rec. A4	inhibitors	rec. tyr. kinase	14572	29
Task7	MUV-692	SF1	agonists	nuclear receptor	14614	30
Task8	MUV-712	HSP90	inhibitors	chaperone	14383	28
Task9	MUV-713	ER-α coact. bind.	inhibitors	PPI	14807	29
Task10	MUV-733	ER-β coact. bind.	inhibitors	PPI	14654	28
Task11	MUV-737	ER-α coact. bind.	potentiators	PPI	14662	29
Task12	MUV-810	FAK	inhibitors	kinase	14615	29
Task13	MUV-832	Cathepsin G	inhibitors	protease	14637	30
Task14	MUV-846	FXIa	inhibitors	protease	14681	30
Task15	MUV-852	FXIIa	inhibitors	protease	14622	29
Task16	MUV-858	D1 rec.	allosteric modulators	GPCR	14745	29
Task17	MUV-859	M1 rec.	allosteric modulators	GPCR	14722	24

Table 3. Data Description of MUV Dataset

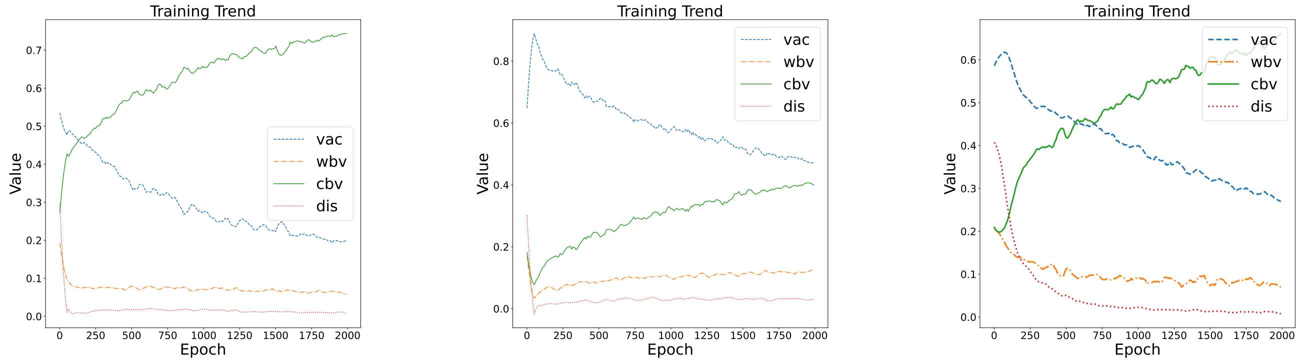

(a) Tox21 (b) SIDER (c) MUV

Fig. 1. Training trend of 10-shot learning with query balancing and AvUC loss.

S3: Additional Results

Learning Curve and Uncertainty Quantification in Training

In the context of model training, several factors play a crucial role in determining the effectiveness of the training process. These factors include the belief vector, vacuity, dissonance, and wrong belief[5]. As shown in 1, the ultimate goal of model training is to minimize dissonance (dis), wrong evidence (wbv), and uncertainty (vac), while simultaneously promoting the emergence of correct evidence (cbv). When these dynamics are accurately depicted in a graph, it signifies that the model has been trained correctly or is well-regularized. By systematically following the training trend, the model becomes more reliable and capable of providing accurate predictions and evidence.

Full Ablation Study Result Including Calibration Errors

Table 4 shows the performance of each variant of our EM3P2.

In addition to the area under the receiver operating characteristic curve (ROC-AUC), we compared the performance of the methods using accuracy (ACC). We also measured the calibration error using two measurements based on test results binned by confidence values (Eq. **??**). The expected calibration error (ECE) are defined as follows [4].

$$ECE = \sum_{m=1}^{M} |B_m|/n|acc(B_m) - conf(B_m)| \quad (1)$$

where $acc(B_m)$ and $conf(B_m)$ are the average accuracy and average confidence for the test data in the mth bin B_m. The ideal result will have both a high accuracy value and a low calibration error. Results with any calibration error value will not matter with low accuracy and vice versa.

Table 4. Ablation studies. Accuracy (ACC) and expected calibration error (ECE) are computed for our EM3P2 variants. QB is query balancing, BR is belief regularizer, and AvUC is accuracy versus uncertainty curve regularizer.

	Dataset		Tox21				Sider				MUV			
	k-shot		1-shot		10-shot		1-shot		10-shot		1-shot		10-shot	
QB	BR	AvUC	ACC	ECE	ACC	ECE	ACC	ECE	ACC	ECE	ACC	ECE	ACC	ECE
✗	✗	✗	0.90	4.0	0.94	4.0	0.64	20.9	0.63	21.3	1.00	-	1.00	-
✓	✗	✗	0.81	4.22	0.83	4.2	0.65	22.4	0.70	22.6	0.82	23.0	0.64	25.4
✓	✓	✗	0.80	15.4	0.81	13.7	0.64	22.9	0.65	21.2	0.78	24.0	0.75	24.2
✓	✓	✓	0.82	13.2	0.82	14.1	0.66	23.0	0.66	21.3	0.80	21.7	0.77	24.2

Effect of Positively Biased and Negatively Biased Tasks on Accuracy vs Calibration Curve

In testing the SIDER dataset, we found that test task 23 resulted in an increase in accuracy as uncertainty increased. To investigate whether this anomaly was due to a mixture of negatively and positively biased tasks, we conducted the following two experiments. First, we used balanced and positively biased tasks to train the metamodel and the negatively biased tasks for testing. The left figure in Fig. 2 shows that when a meta-model is trained with negatively biased tasks, testing the model with positively biased tasks confuses the uncertainty values.

Second, we reversed the labels of the negatively biased items so that all items were positively biased. The right plot in Fig. 2 shows the results. We can see that making all the items one-sided helps to improve accuracy as uncertainty decreases. However, task 23 (T23) still has a low overall accuracy. We suspect that this is due to the fact that Task 23 is the side effect category for "pregnancy, puerperium & perinatal conditionsholds", which is a significantly different categorization than most of the other tasks that map side effects to system organ classes.

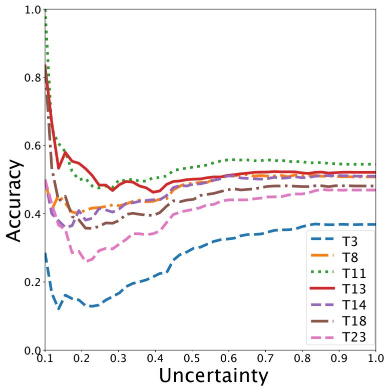

(a) Tox21

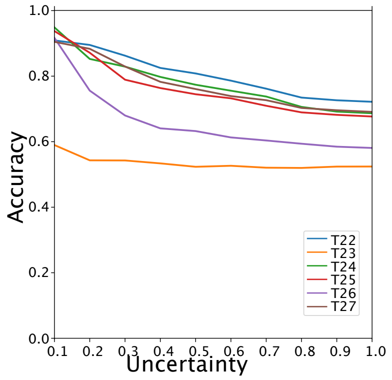

(b) SIDER

Fig. 2. Accuracy vs Uncertainty for two tests in SIDER.

Additional Empirical Studies

Structure of captafol (left) and oxymetholone (right) for MPP prediction evaluated for Tables 5 (main text) and Table S5 and S6.

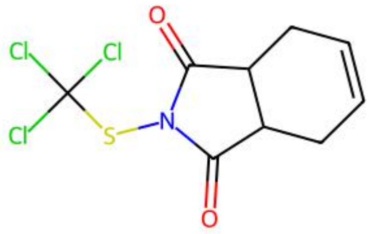

(a) Tox21

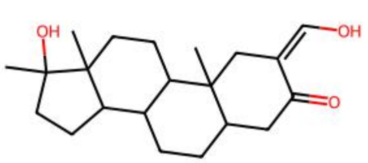

(b) SIDER

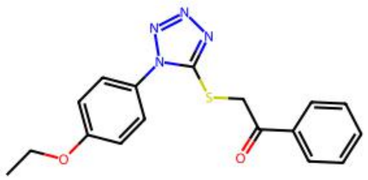

(c) SIDER

Fig. 3. Structure of captafol (left) and oxymetholone (center) Compound CID: 659783 MF: C17H16N4O2S (right) for MPP prediction evaluated for Tables 5 and 6.

Table 5 shows the prediction results for oxymetholone in the SIDER dataset. The true label was all negative for the six test tasks. Again, the MLP-based prediction predicted wrong with a very high class probability, while our EM3P2 using EMLP predicted 'I don't know (?)'.

Table 6 shows the prediction results for Compound CID:659783 in the MUV dataset. The true label was all positive for the three test tasks. Again, the MLP-based prediction predicted wrong with a very high class probability, while our EM3P2 using EMLP predicted 'I don't know (?)'.

Table 5. Detailed test task result of oxymetholone in SIDER dataset.

	Measure	Task22	Task23	Task24
EM3P2	+ Evidence	0.063	0.090	0.103
	Uncertainty	0.885	0.880	0.868
	Prediction	?	?	?
EM3P2 (MLP)	+ Class prob.	0.948	0.953	0.963
	Prediction	+	+	+
	Measure	Task25	Task26	Task27
EM3P2	+ Evidence	0.010	0.080	0.068
	Uncertainty	0.867	0.876	0.865
	Prediction	?	?	?
EM3P2 (MLP)	+ class prob.	0.967	0.979	0.976
	Prediction	+	+	+

Table 6. Detailed test task result of C17H16N4O2S in MUV dataset

	Measure	Task13	Task16	Task17
EM3P2	+ Evidence	0.075	0.043	0.068
	Uncertainty	0.603	0.501	0.533
	Prediction	?	?	?
EM3P2 (MLP)	− Class prob.	0.986	0.997	0.997
	Prediction	−	−	−

References

1. Chelsea Finn, Pieter Abbeel, and Sergey Levine. Model-agnostic meta-learning for fast adaptation of deep networks. In *International conference on machine learning*, pages 1126–1135. PMLR, 2017.
2. Weihua Hu, Bowen Liu, Joseph Gomes, Marinka Zitnik, Percy Liang, Vijay Pande, and Jure Leskovec. Strategies for pre-training graph neural networks. *The International Conference on Learning Representations (ICLR)*, 2020.
3. Gregory Koch, Richard Zemel, Ruslan Salakhutdinov, et al. Siamese neural networks for one-shot image recognition. In *ICML deep learning workshop*, volume 2, page 0. Lille, 2015.
4. Mahdi Pakdaman Naeini, Gregory Cooper, and Milos Hauskrecht. Obtaining well calibrated probabilities using bayesian binning. In *Proceedings of the AAAI conference on artificial intelligence*, volume 29, 2015.
5. Deep Shankar Pandey and Qi Yu. Multidimensional belief quantification for label-efficient meta-learning. In *Proceedings of the IEEE/CVF Conference on Computer Vision and Pattern Recognition*, pages 14391–14400, 2022.
6. Jake Snell, Kevin Swersky, and Richard Zemel. Prototypical networks for few-shot learning. *Advances in neural information processing systems*, 30, 2017.
7. Yaqing Wang, Abulikemu Abuduweili, Quanming Yao, and Dejing Dou. Property-aware relation networks for few-shot molecular property prediction. *Advances in Neural Information Processing Systems*, 34:17441–17454, 2021.
8. Oliver Wieder, Stefan Kohlbacher, Mélaine Kuenemann, Arthur Garon, Pierre Ducrot, Thomas Seidel, and Thierry Langer. A compact review of molecular property prediction with graph neural networks. *Drug Discovery Today: Technologies*, 37:1–12, 2020.
